# Intraoperative practices to prevent wrong-level spine surgery: a survey among 105 spine surgeons in the United Kingdom

**DOI:** 10.1186/s13037-021-00310-9

**Published:** 2022-01-26

**Authors:** Ali Zain Naqvi, Henry Magill, Naffis Anjarwalla

**Affiliations:** 1 Orthopaedic Registrar, St Mary’s Hospital, London, UK; 2 Orthopaedic Registrar, Chelsea and Westminster Hospital, London, UK; 3 Consultant Orthopaedic Surgeon, Wexham Park Hospital, Slough, UK

## Abstract

**Background:**

Current literature suggests that wrong-level spine surgery is relatively common with far-reaching consequences. This study aims to assess the current practices of spinal surgeons across the UK with respect to the techniques implemented for correct level verification.

**Methods:**

To assess the current practices of spinal surgeons across the UK with respect to the techniques implemented for level verification. The authors hypothesise the absence of a standardised technique used across spine surgeons in the UK. Practices amongst respondents will be ascertained via an electronic questionnaire designed to evaluate current practices of spinal surgeons whom are members of the British Association of Spinal Surgeons (BASS). The study data will include key information such as; the level of surgical experience, specific techniques used to perform level checks for each procedure and prior involvement with wrong-level spine surgery. Responses were collected over the period of 1 month with a reminder sent 2 weeks prior to closure of the survey. The data were collated and descriptive analyses performed on multiple-choice question answers and common themes established from free text answers.

**Results:**

A total of 27% (*n* = 105/383) members responded. The vast majority had greater than 10 years’ experience. Intraoperative practices varied greatly with varying practices present for cervical, thoracic and lumbar level surgery. Only 38% (*n* = 40) of respondents re-checked the level intra-operatively, prior to instrumentation. Of the respondents 47.5% (*n* = 29/61) of surgeons had been involved in wrong level spinal surgery.

**Conclusion:**

This study highlights the varying practices amongst spinal surgeons and suggests root cause for wrong-level spine surgery; where the level identified pre-incision was subsequently not the level exposed. We describe a novel safety-check adopted at our institute using concepts and lessons learnt from the WHO Checklist.

## Background

“Never events” have been conceptualised in the NHS since 2009, and are described as patient safety incidents that are considered preventable [1]. Initially there were eight adverse patient safety events of which one was *wrong-site surgery*; this encompassed surgical intervention performed on the wrong patient or wrong site [2].

Despite substantial efforts “never events” have been persistent within health care; in the United Kingdom national standards were introduced for patients undergoing invasive procedures in 2012 and these culminated in NHS England launching the national safety standards for invasive procedures (NatSSIPs) [3]. These standards have been introduced alongside checklists such as the World Health Organization (WHO) surgical safety checklist and the five steps to safer surgery [4, 5]. The use of a pre incision checklist was felt to improve patient safety by 98.9% of the members of a surgical team in a neurosurgical centre in a study by Mclaughlin et al. [6]. Checklist advantages have been studied recently in a Chinese nationwide survey where it found to be a powerful tool in improving patient safety [7]. Further to this, a Swiss study considered factors relating to adherence to the “time-out” and “sign out” checklists; Cushley et al. demonstrated increased engagement with adaptations made to the way the checklist was carried out [8, 9].

Even in the presence of validated checklists with engagement from the surgical team, there has not been a significant reduction in the occurrence of “never events” where wrong-site surgery is still prevalent. Wrong-site surgery is accountable for 138 cases of the 344 “never events” reported by trusts across England between April 2018 and November 2018 [10]. Wrong-level spine surgery remains a significant event and the consequences of this are far reaching with possible effects to patient’s health as well as the confidence of the operating surgeon and the team. In addition, there are significant cost implications associated with wrong-level spine surgery; a national report in 2011–2012 disclosed that the NHS paid out £137 million on orthopaedic negligence cases alone [11].

Current literature and case studies have suggested that wrong-level spine surgery is a relatively common error whilst performing spinal surgery, where it predominantly occurs a level above the intended level [12]. A large variation of quoted rates exist within literature; where some estimations suggest that 1 in 2 spine surgeons will perform at least one wrong-level spine surgery in their career [13, 14]. With this in mind, we have designed a survey to gauge the current practices of the spine surgeons who are members of British Association of Spinal Surgeons (BASS). The aim of this study was to investigate if consensus existed with respect to level checks for spine surgery and highlight any outlying methods. Using the responses received we then aim to describe a novel method that we have adopted at our institute using concepts and lessons learnt from the WHO Checklist.

## Methods

Spinal surgery is unique in that it regularly requires a level check. The authors hypothesise that level checks are often ascertained in a non-standardised method with a significant proportion of spine surgeons still having involvement in wrong-level spine surgery. A questionnaire was designed to evaluate current practices of spinal surgeons with membership to BASS to obtain current practises amongst spinal surgeons. Questions were designed to evaluate:

- Level of surgical experience
- Current technique used to perform level checks
  - Specific responses for cervical, thoracic and lumbar procedures
- Prior involvement with wrong-level spine surgery and lessons learnt

**Fig. 1:**
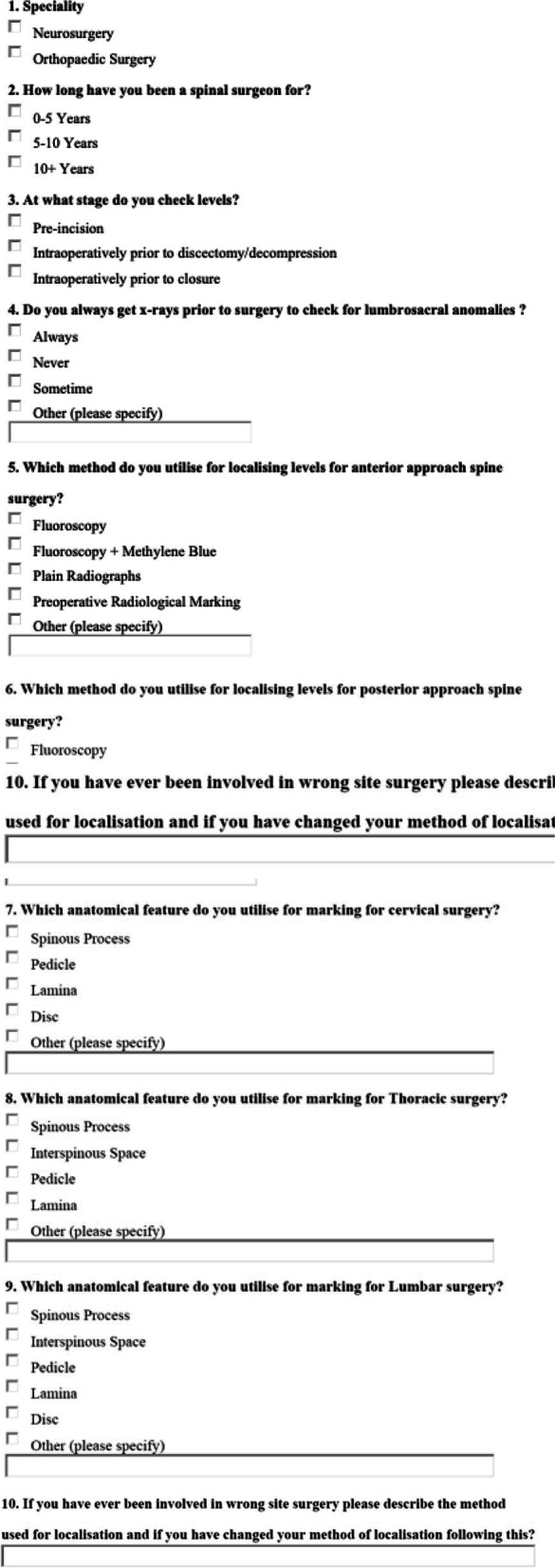
The ten-question survey

A survey consisting of 10 questions was developed with responses in multiple-choice format and free text (Fig. 1). All respondents had the option to elaborate in the free text area if required. For surgeons not performing certain procedures an option to select *“Not applicable”* was available.

Invitations to complete the survey were sent to all 383 members of the BASS by electronic mail beginning of March 2018. A link was provided within the electronic mail to a web interface to facilitate data collection and no personal data was required ensuring confidentiality was maintained. One subsequent reminder was sent via electronic mail 1 month later, with no further requests thereafter.

All multiple-choice responses were collated and descriptive analyses performed. Free text responses were categorised by recurring themes.

## Results

Over the course of 1 month, 27% (*n* = 105/383) of BASS members responded. A total of 79 (75.2%) responders were orthopaedic spine surgeons; where 26 (24.8%) were neurosurgeons. A small majority, 60% (*n* = 63) of surgeons had greater than 10 years’ experience. Of the 105 respondents, 60% (*n* = 63) performed checks prior to incision and 38.1% (*n* = 40) rechecked intra-operatively prior to decompression, discectomy or screw insertion, with only 1.9% (*n* = 2) checking prior to closure.

**Fig. 2:**
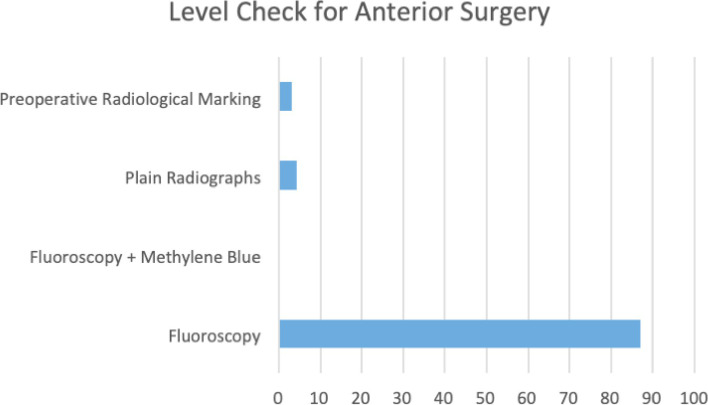
The method by which the level check was performed for anterior surgery

**Fig. 3:**
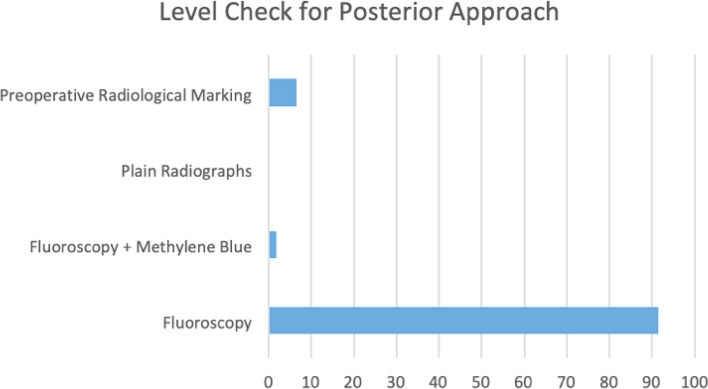
The method by which the level check was performed for posterior surgery

There was overall consensus with 86.7% (*n* = 91) and 92.4% (*n* = 97) using fluoroscopy for additional preoperative marking, for anterior (Fig. 2) and posterior (Fig. 3) approaches to the spine respectively. There appears to be a large variability in practice when anatomical landmarks of choice are considered; 75.2% (*n* = 79) of those carrying out cervical spine surgery reported using the disc space as their primary landmark of choice. However, there was significant heterogeneity when using landmarks in in thoracic and lumbar procedures. In thoracic surgery, 48.6% (*n* = 51) of respondents used pedicles as their primary landmark, 17.1% (*n* = 18) used spinous processes and the remainder primarily using various other landmarks including laminae, disc spaces, interspinous processes or ribs.

A similar pattern existed with lumbar spine surgery with respect to use of landmarks. Disc spaces were used as the primary landmark of choice by only 41.9% (*n* = 44) of those carrying out lumbar surgery. Interspinous space, laminae, pedicles, spinous process and facets were all used as the primary anatomical landmark for lumbar surgery by the remaining respondents with 19.0% (*n* = 20), 16.2% (*n* = 17), 12.4% (*n* = 13), 10.5% (*n* = 11) and 0.9% (*n* = 1) respectively. The survey also revealed lumbosacral anomalies are not routinely checked by those involved in the survey with only 28.6% (*n* = 30) routinely getting radiographs preoperatively to specifically check for abnormalities of the lumbosacral spine.

Lastly, involvement in wrong-level spine surgery and lessons learnt was analysed. This question was answered by 61 of the 105 total respondents with 47.5% of those who responded (*n* = 29) having been involved either directly or indirectly with such an event. *Direct involvement* was participation as the primary operating surgeon. On analysis of additional comments by those involved in wrong-level spine surgery, common themes have emerged.

The main alteration in practice following wrong-level spine surgery is the timing of lateral images; where 27.6% (*n* = 8) of involved surgeons now describe the use of image intensifier prior to incision. Such practices involve the placement of a radiopaque object and obtaining images to confirm the level prior to proceeding. Another theme is of the misinterpretation of the imaging or the presence of transitional vertebrae with 6.9% (*n* = 2) of involved surgeons describing incidences of wrong-level spine surgery pertaining to these factors.

## Discussion

Wrong-level spine surgery is multifactorial and even with the aide of intraoperative imaging this “never event” may still occur [15]. Risk reduction strategies have been suggested including standardised protocols to prevent wrong-level spine surgery [16]. A number of patient deaths have previously been attributable to wrong site surgery; a root cause analysis in a recent study revealed 72% of wrong-site surgery occurred due to the lack of a “time-out” [17]. Wrong-level spine surgery was found to be more prevalent with the absence of a “time-out” where 60% of surgeons in one study did not use intraoperative imaging in their practices [18]. Considerable efforts have been made into the development of spine specific checklists however, not one is routinely utilised universally [19].

In our survey, certain preoperative investigations may aid in the reduction of possible wrong-level spine surgery. Anatomical variations can compound an already significant problem where it has been suggested that an anatomical variation of 11 ribs is present in 3.4% of the population [20]. Furthermore, the presence of cervical ribs may also need to be accounted for, however, obtaining plain radiographs was not routine practice by the vast majority of surgeons responding to our survey [21].

Although some areas of practice showed significant consistency, with a large majority (> 80%) using fluoroscopy as the mainstay of level checks for both anterior and posterior spinal surgery. Specific fluoroscopy techniques and timings varied greatly with all approaches. Large variation existed in the use of landmarks and whether the checks were pre-incision, pre-instrumentation or pre-closure. This heterogeneity was particularly apparent in thoracic and lumbar surgery. The individual practices implemented showed no correlation with years of experience or speciality. Most interestingly, almost half of the surgeons have been involved with wrong-level spine surgery in some form.

Laxer et al. state that, despite the use of these site verification protocols, the reported number of wrong-site sentinel events have continued to increase [8]. The authors of this study therefore propose an alternative method of level check confirmation, similar to the WHO “time-out”. A specific safety pause prior to instrumentation, involving the entire theatre team, is implemented. Similar to the WHO “time-out”, the method involves a degree of shared responsibility and multi-disciplinary involvement. Such level checks can be performed regardless of the technique implemented and should involve the anaesthetist, radiographer and surgeon reviewing the intensified images and the placement of a radiopaque marker at the intended level. Any doubt, disagreements or anatomical variation should be given due consideration through a natural pause and discussion where required. The concept of shared responsibility and team involvement is the premise of our proposed safety pause. A similar model was tested with surgical site marking in Switzerland which resulted in 0% wrong-site surgery, which was maintained over a 2 year period [22].

A standardised post-exposure, pre-instrumentation safety check would therefore eliminate possible “never events”. Challenges will be encountered and consideration must be given to the effect of adaptations being made on the surgical team and the readiness with which it is adopted by surgical teams [23–25].

The authors of this study appreciate the limitations of this study. Primarily, a total of 105 respondents are unlikely to give an entirely accurate representation of the spinal practises across the United Kingdom. Despite the anonymity of the collected responses, we also appreciate the collected questionnaire data may not wholly reflect the current practises or previous involvement in wrong-level spinal surgery.

## Conclusion

Large variability is present amongst level checks amongst spinal surgeons. This survey has shown the absence of a standardised technique with on-going involvement in wrong-level spinal surgery by a significant proportion of surgeons. A possible root cause for wrong-level spine surgery has possibly been highlighted where the level identified pre-incision was subsequently not the level exposed. To eliminate heterogeneity we describe a novel and standardised post-exposure, pre-instrumentation safety check utilised in our institute using concepts and lessons learnt from the WHO Checklist and guided by responses obtained from members of BASS.

## Data Availability

Not applicable.

## Abbreviations

NHS: National Health Service
UK: United Kingdom
WHO: World Health Organisation
BASS: British Association of Spinal Surgeons
